# A metameric origin for the annelid pygidium?

**DOI:** 10.1186/s12862-015-0299-z

**Published:** 2015-02-25

**Authors:** Viktor V Starunov, Nicolas Dray, Elena V Belikova, Pierre Kerner, Michel Vervoort, Guillaume Balavoine

**Affiliations:** Department of Invertebrate Zoology, Saint-Petersburg State University/ Universitetskaya nab. 7/9, 199034 Saint-Petersburg, Russia; Zoological Institute RAS/ Universitetskaya nab. 1, 199034 Saint-Petersburg, Russia; Institut Jacques Monod, CNRS/Université Paris Diderot, 15 rue Hélène Brion, 75013 Paris, France; Institut Universitaire de France, Paris, France

**Keywords:** Polychaeta, Pygidium, Segmentation, Origin of metamerism, Cyclomeric theory, Colonial theory, *Platynereis dumerilii*

## Abstract

**Background:**

Segmented body organizations are widely represented in the animal kingdom. Whether the last common bilaterian ancestor was already segmented is intensely debated. Annelids display broad morphological diversity but many species are among the most homonomous metameric animals. The front end (prostomium) and tail piece (pygidium) of annelids are classically described as non-segmental. However, the pygidium structure and development remain poorly studied.

**Results:**

Using different methods of microscopy, immunolabelling and a number of molecular markers, we describe the neural and mesodermal structures of the pygidium of *Platynereis dumerilii*. We establish that the pygidium possesses a complicated nervous system with a nerve ring and a pair of sensory ganglia, a complex intrinsic musculature, a large terminal circular blood sinus and an unusual unpaired torus-shaped coelomic cavity. We also describe some earlier steps of pygidial development and pygidial structure of mature animals after epitokous transformation.

**Conclusions:**

We describe a much more complex organization of the pygidium of *P. dumerilii* than previously suggested. Many of the characteristics are strikingly similar to those found in the trunk segments, opening the debate on whether the pygidium and trunk segments derive from the same ancestral metameric unit. We analyze these scenarios in the context of two classical theories on the origin of segmentation: the cyclomeric/archicoelomate concept and the colonial theory. Both theories provide possible explanations for the partial or complete homology of trunk segments and pygidium.

## Background

*“The pygidium may or may not represent single somites”*

Huxley T. H. – lectures on general Natural History – lectures VI, Medical Times and Gazette, Vol xiii, N. S., 1856

*“The only dogmatic statement we are justified in making is, that when a region exhibits during development a sufficient number of the essential structures of a typical segment, it may be assumed to be a true metamere. What is “sufficient” has to be decided in each case”*

Goodrich E.S. – Memoirs: On the Relation of the Arthropod Head to the Annelid Prostomium. –Quarterly Journal of Microscopical Science 1897, 40: 247–268.

The origin of metameric segmentation is a key issue on the way to understanding how the vast diversity of bilaterian or triploblastic animals evolved. There is currently no consensus on the question, as two incompatible views are in competition: either segmentation of the body axis evolved once in the early history of metazoans and the last common ancestor of bilaterian animals (commonly referred to as "*Urbilateria*") was indeed a metameric animal [1-3], or non-homologous segmentations evolved independently in several metazoan lineages [4-6]. Defenders of both views have advanced valuable arguments in recent years. On one side, the overall picture of current knowledge seems to suggest that the ways segments are made in distant groups of segmented bilaterians, such as insects and vertebrates, are very divergent at morphogenetic, cellular and genetic levels. On the other side, intriguing similarities have nevertheless been found in the way segments are patterned in distant unrelated bilaterian groups, such as the proposed involvement of the Notch pathway in segment formation in arachnids [7], centipedes [8] and at least some insects [9] in a way that is reminiscent of its role in vertebrate somitogenesis, or the discovery of genes in annelids that are expressed in segment polarity-like patterns as their orthologues are in arthropods [10-13].

The group of bilaterians that most strikingly matches an idealized definition of metameric body organization from an adult morphology point of view are the annelids [14]. Annelids are an ecologically important group that exists at least since the Cambrian and are extremely diverse in terms of body shapes. Some have even lost segmentation altogether: echiurids and sipunculids, until recently considered phyla on their own right, have been convincingly shown to be derived annelid groups [15-19]. The ancestral morphology of annelids is proposed to include a combination of characters found among the numerous families of “polychaetes” [16,19]. The body of this presumably ancestral annelid is formed of a head, trunk and “tail end” or pygidium. The head is classically considered to be formed of the prostomium, bearing the cerebral ganglia and sense organs, and the peristomium, a segment-like structure bearing the mouth and occasionally sensory cirri. The trunk of the sexually immature worm is a succession of nearly identical segments (homonomous segmentation), although graded variations of the organ morphologies are visible in anterior segments. In most annelid species, a number of anterior segments are produced during larval life but posterior elongation continues during post-larval benthic juvenile development. Segments are produced sequentially by a budding process just in front of the pygidium, at the level of a segment addition zone (SAZ) [20,21] during most of the life of the animal. The pygidium is the terminal piece bearing the anus and in many groups, sensory cirri. During larval development, the prostomium originates from the larval episphere, the peristomium, larval body segments and SAZ emerge from the anterior part of the hyposphere, and the posterior part of the hyposphere gives rise to the pygidium [22,23].

The prostomium and the pygidium have always been presented as “non-segmental” in nature as authors usually adopt an “idealistic” or “typological” view of segmentation. In summary, a structure is considered a segment if it groups together an outer annulus, one pair of mesodermal coelomic cavities, one pair of ventral ganglia, a set of muscles, one pair of appendages and one pair of metanephridia [24]. However, during evolution, organs and structures often change form and function and are sometimes present as derived or even rudimentary structures. The general question that we ambition to start addressing in this work is the evolutionary origin of the pygidium. A useful analogy can be drawn with the much more consensual evolutionary history of the tetrapod limb: strictly speaking, a human arm does not have the characters that would allow classifying it as a “fin” but, from a large amount of data coming from the anatomy, paleontology and developmental biology fields, we deduce that human arms must have originated from the anterior paired limbs of a fish-like ancestor, therefore from “fins”. Assessing the resemblance of the pygidium to trunk segments however requires a careful structural analysis, which was not available to date. Brief descriptions of external pygidial morphologies for each annelid family are available in recent polychaete handbooks [25,26]. In most families, the pygidium is a small and inconspicuous more or less rounded piece, often bearing one or two pairs of sensory cirri. In some families, it is simple without cirri. In a few species, a larger number of pygidial cirri are found. The external pygidial morphology has been better described only in a few groups for taxonomic purposes (Spionids or Maldanids [27,28]). The internal morphology of this body region has been barely addressed [29]. For annelids as for all other animals, the “trivial” tail end has attracted much less attention than the “noble” head part.

The family Nereididae stands as one that presents many of the anatomical characteristics of the hypothesized ancestral annelid [14]. *Platynereis dumerilii* has become in recent years a model organism for developmental biology as well as for other research fields [30]. In this work, we explore in depth the neural and mesodermal structures of the pygidium of *P. dumerilii*, with new descriptive data using electron microscopy and a number of molecular markers combined with confocal microscopy. We also follow the poorly explored ontogeny of the pygidium. We describe a much more complex organization than previously suggested with a true unpaired coelomic cavity, a complex intrinsic musculature, a nerve ring and a pair of sensory ganglia. Many of these characteristics resemble those found in the normal segments of *P. dumerilii*. The pygidium is therefore not far from the typological definition of a metamere. More important, however, than this strict definition issue, we discuss what these new data imply for the origin of the pygidium, i.e. that it may share a common evolutionary origin with the trunk segments. We analyse this idea in the context of the evolutionary theories of the emergence of segmentation in annelids and in bilaterians.

## Results

### Brief description of the pygidium development in *P. dumerilii*

**Figure 1:**
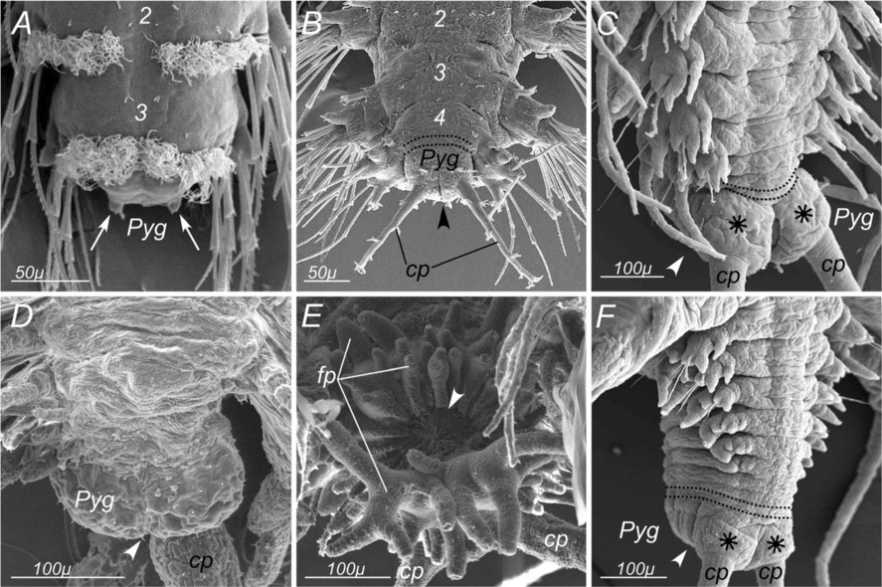
**Scanning electron micrographs of** ***P. dumerilii*** **pygidium at different stages of the life cycle. A**: pygidium of the 3-day larva, view from the dorsal side. Arrows show developing pygidial cirri. **B**: pygidium of the 4-segmented larva, view from the dorsal side. **C**: posterior part of the young atokous worm, view from the ventral side. **D**: dorsal view of epitokous female worm pygidium. **E**: pygidium of epitokous male worm, view from the dorsal side. **F**: posterior part of the worm after caudal regeneration, view from the dorsal side. Numbers in **A** and **B** mark chaetigerous segments in the antero-posterior direction. Arrowheads point the position of the anus. Asterisks show bases of pygidial cirri. Dotted lines show the borders of the segment addition zone. *Pyg* – pygidium; *cp* – pygidial cirri; *fp* – finger-like protrusions of mature male pygidium.

The embryonic, larval and post-larval development of *P. dumerilii* has been described in details elsewhere [31]. Embryogenesis gives a microscopic spherical larva that transforms after three days into a segmented larva with three chaetigerous (bearing chaetae) segments. The pygidium is far from fully formed at this stage. It first appears as a bulge on a 3-day larva posterior end (Figure 1A) with buds of a pair of pygidial tentacular cirri (Figure 1A, arrows). Shortly after, the bulge elongates and the cirri grow rapidly (Figure 1B). A functional anus is probably not present before 10 days, when the previously lecithotrophic larva starts to feed. At the same time as the pygidium takes shape, a segment addition zone (SAZ) differentiates just in front of the pygidium and the fourth chaetigerous segment rudiment progressively appears (Figure 1B). The pygidium continues to grow in size as the juvenile worm adds more segments. In particular, a pair of voluminous glands develops ventrally at the base of the tentacular cirri (Figure 1C). The pygidium thus clearly differentiates after the three first anterior-most segments but before all the remaining trunk segments.

At the end of the life cycle, a dramatic sexual metamorphosis (*heteronereis* form) divides the trunk in two tagmata, the segments of the posterior part turning into a swimming organ. The pygidium also undergoes significant changes in its shape. These changes are more pronounced in males than in females (Figure 1D, E).

*P. dumerilii* is capable of caudal regeneration. After an amputation of the posterior half of the body, a regeneration blastema quickly forms. A new anus appears and two cirri bud. Then a new SAZ starts to function in front of the new pygidium (Figure 1F). Clear differences in the precise timing of events thus occur between the formation of the pygidium in late larval development and regeneration. Therefore, we studied the morphology of pygidium in the atokous worm as found in a four month-old sub-adults, in the epitokous worm, and also the newly regenerated pygidium, as found in sub-adults seven to ten days after posterior amputation.

We concentrated our work on those structural elements that are found in the segments of the vast majority of annelid groups and are all present in the segments of *P. dumerilii* thus allowing direct comparison with similar structures in the pygidium. We found a number of structures that resemble these segmental elements in the mature and forming pygidium.

### A transient telotroch is present on the *P. dumerilii* forming pygidium

**Figure 2:**
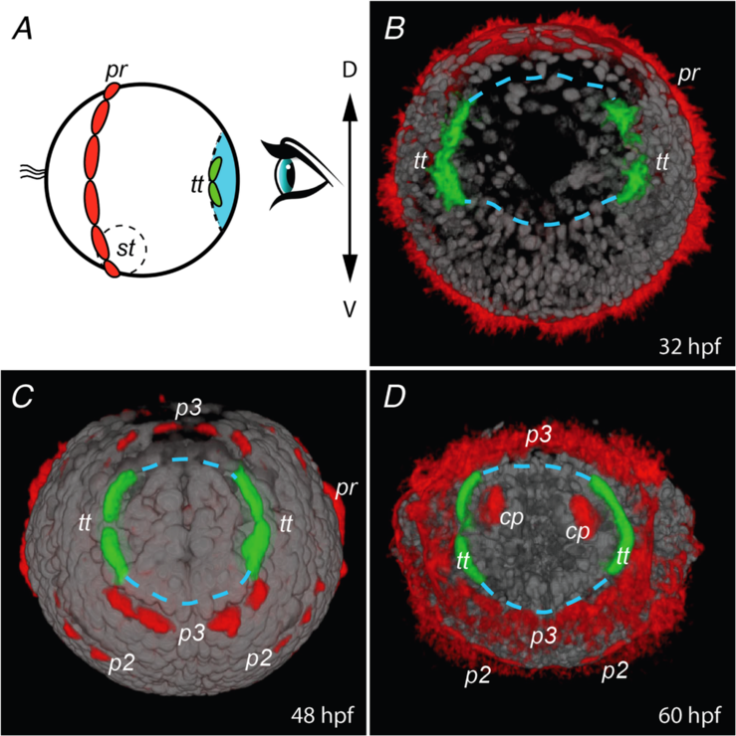
**Posterior ciliary belts of the** ***P. dumerilii*** **larva.** Bottom views of developing larvae showing the changing region of the future pygidium. Nuclei are stained with DAPI (grey) and cilia with an anti-acetylated tubulin antibody. Views are 3D renderings of confocal stacks of images, made with Image J. The telotroch cilia have been false-colored in green whereas prototroch and paratroch cilia are colored in red. **A**: schematic representation of the perspective chosen for the 3D rendering, the presumptive pygidial territory is colored in cyan. **B-D**: 3D reconstructions of larvae at 30, 48 and 60 hpf, respectively. The presumptive pygidium is delineated with blue dashed lines. *pr* – prototroch; *p2* and *p3* – paratrochs of the second and third chaetigerous segments respectively; *tt* – telotroch; *st* – stomodeum; *cp* – pygidial cirri.

In addition to the well-known prototroch found in the larvae of many trochozoans, each early larval segment in many annelids bears a belt of multiciliated cells called paratroch. These belts are involved in ciliary swimming until the larva selects a place to settle on the sea floor. Paratrochs, as well as the prototroch, then usually degenerate and are not observed in growing juveniles. Many annelid larvae bear also a telotroch, located posterior to the segment addition zone and which therefore belongs with the pygidium [22]. In previous work on nereidid development, a telotroch has been described in the early trochophore (for example [31]), developing before the paratrochs. We used an anti-acetylated alpha-tubulin antibody to label the cilia and followed more precisely the development of the larva of *P. dumerilii* multiciliated cell apparatus. The telotroch appears in the early trochophore stage as a bilateral pair of crescent-shaped ciliary structures, surrounding the future pygidial ectoderm (Figure 2B, *tt*). In the late trochophore stage, the paratroch of the third segment also appears nearby in a complementary pattern to the telotroch, ventrally and dorsally (Figure 2C, *p3*). The two structures form together a complete ciliary belt that possibly functions as a single locomotory unit at this stage (Figure 2C, *p3 + tt*). As the larva elongates, the pygidium starts to take shape. In a 60 hour old nectochaete, a diminutive but distinct telotroch persists as two small groups of multiciliated cells located on each side, anteriorly in the pygidium (Figure 2D, *tt*). The telotroch, paratrochs and prototroch gradually disappear after the larva has settled on the substrate.

### Nervous system and sensory organs

**Figure 3:**
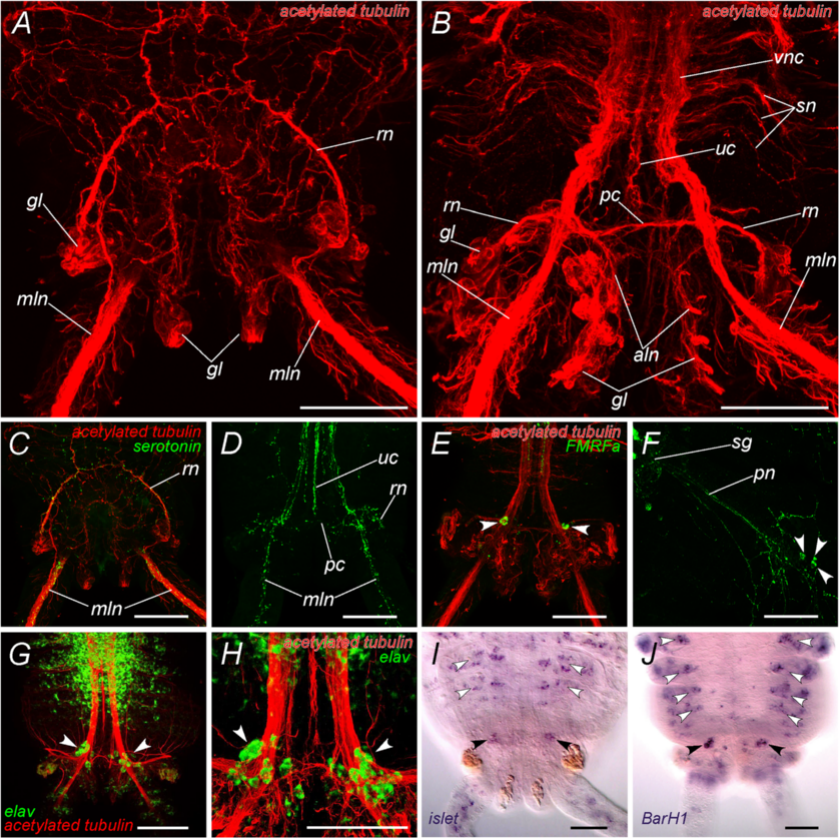
**Molecular characterization of the nervous system of atokous** ***P. dumerilii*** **pygidium. A-B**: Confocal micrographs showing acetylated α-tubulin immunoreactivity. **C-D**: Serotonin-positive (green) and acetylated α-tubulin immunoreactivity (red, **C** only). **E-F**: FMRFamide-like (green) and acetylated α-tubulin immunoreactivity (red, **E** only) in pygidium **(E)** and the peripheral nerves of a mid-body parapodium **(F)**. **G-H**: *elav*-positive cells in the pygidium (green); nerves are counterstained with acetylated α-tubulin antibodies (red). **I**: *Pdu-Islet* expression in the pygidium and nascent segments. **J**: *Pdu-BarH1* expression in the pygidium and nascent segments. View from the dorsal **(A, C)** and from the ventral **(B, D-J)** sides. *sn* - developping segmental nerves; *vnc* - ventral nerve cord; *uc* - unpaired nerve of the ventral nerve cord; *rn* - pygidial ring nerve; *pc* - pygidial commissure; *mln* - main longitudinal nerve; *aln* - additional pygidial longitudinal nerves; *gl* - pygidial glands; *pn* - parapodial nerve. Arrowheads show FMRFamide-like immunoreactive cells **(E, F)** and *elav-*positive cells **(G, H)**.Black arrowheads and white arrowheads show *Islet-* **(I)** and *BarH1-* **(J)** positive cells in the pygidium and in cells of the parapodial ganglia, respectively. Scale bar 50 μm.

The fully developed pygidium of the atokous worm bears on its ventral side two pairs of glands which are extremely autofluorescent, making the study of its morphology with epifluorescent microscopy techniques challenging. However, this problem is limited when immunochemical staining procedures are performed on the newly regenerated pygidium of worms that have been amputated at least 7 days before. Antibodies against acetylated α-tubulin reveal that the pygidium possesses two main ventral longitudinal nerve cords, which are the terminal parts of the trunk ladder-like nerve chain (*mln,* Figure 3A, B). The nerve cords pass through the pygidium and each one extends in one of the pygidial cirri. A number of tiny nerves emanates from these cords to innervate the surface of cirri and pygidial glands. The unpaired small nerve of the ventral nerve cord can be followed only to the border between pygidium and the last body segment (*uc,* Figure 3B). In the anterior part of the pygidium a conspicuous nerve ring is seen (*rn,* Figure 3A, B). It branches from the main longitudinal cords. At the same longitudinal level, a tiny commissure interconnects longitudinal cords (*pc,* Figure 3B). The ring nerve is connected by a multitude of small neurites with the peripheral nerve net of the body wall as well as to the gut nerve plexus.

Serotonin-positive immunoreactivity was detected in all main parts of pygidial nervous system including pygidial commissure and ring nerve (Figure 3C, D). At the ventral side serotonin-positive neurites form two little plexuses associated with glands. No serotonin-positive perikaria were found in the pygidium.

FMRFamide-like immunoreactivity, on the contrary, is very scarce. Almost no sign of FMRFamide-like neurites were found neither in main longitudinal nerves, nor in the ring nerve and pygidial commissure. However on the ventral side of the pygidium at the level of the “main branching points” two FMRFamide-like immunoreactive cells were found (Figure 3E, arrowheads). These neurons are very similar to those found in parapodial ganglia of normal trunk segments (Figure 3F). To find out whether there are more neurons in this place, we labeled differentiating post-mitotic neuron precursors during post-caudal regeneration elongation with a *Pdu-elav* probe, a differentiating neuron marker, using whole-mount in situ hybrization (WMISH) [32]. Double labeling for *Pdu-elav* and acetylated alpha-tubulin thus allows seeing most of the differentiating nervous system of the posteriorly growing juveniles (Figure 3G, H) after caudal regeneration. On the ventral side of the pygidium, surrounding the main nerve branching points, two small groups of presumptive neurons are revealed by the *elav* staining (Figure 3G, H, white arrows). They are much smaller than the ganglia of the ventral nerve cord, comprising only a dozen visible cells each. To get some insight into the identities and functions of these cell groups and to determine whether they could be homologous to cells of the segmental ganglia, we analyzed by WMISH the expression of a whole series of neural differentiation genes. In general, we found that markers that are specifically associated with the ganglia of the ventral nerve cord (*NK1, HB9, Coe*) are not expressed in these small pygidial ganglia [11,33,34]. We found instead clear expressions of *Islet* and *BarH1* in all or part of the neurons of the pygidial ganglia (Figure 3I, J, black arrowheads). These two markers are specifically associated with the segmental peripheral ganglia (Figure 3I, J, white arrowheads and Béhague, Kerner, et al., in preparation). This suggests that the two small pygidial ganglia contain neurons with the same molecular identities as at least some neurons of the parapodial ganglia of the trunk and could represent homologous, presumably sensory, neuronal types.

### Muscular system

**Figure 4:**
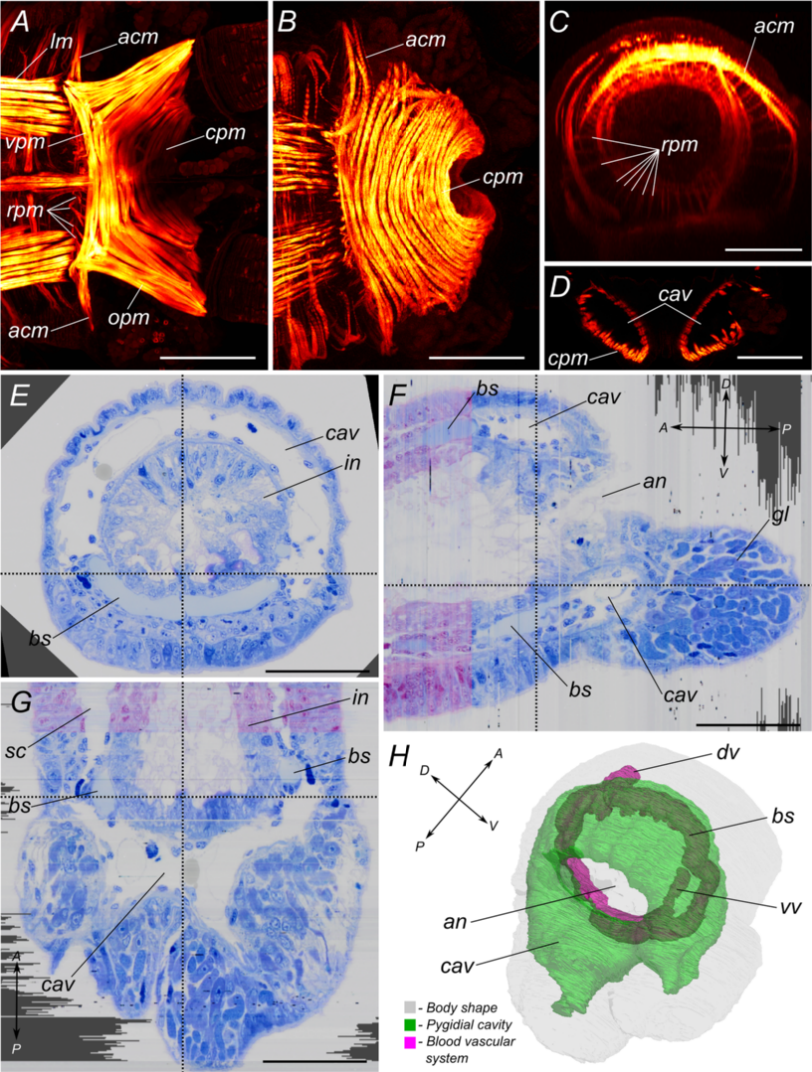
**Muscular system of atokous** ***P. dumerilii*** **pygidium and reconstruction of pygidial coelomic cavity. A-D**: Confocal micrographs showing TRITC-phalloidin labelled muscles in the posterior end of the worm. **A**: view from the ventral side; **B**: view from the dorsal side; **C**: virtual cross-section at the level of the border between pygidium and segment addition zone; **D**: an optical longitudinal section showing the position of pygidial cavity between body wall musculature and hindgut muscular lining. **E-G**: Orthogonal sections through the series of semithin slices of the pygidium. **E**: cross-section; **F**: virtual sagittal section; **G**: virtual longitudinal section. **H**: 3D-reconstruction of the pygidial cavity and perianal blood sinus. Dotted lines in **E-G** mark planes of virtual sectioning. *acm* – additional circular muscle; *an* – anus; *bs* – blood vessels; *cav* – pygidial cavity; *cpm* – circular pygidial muscle fibers; *dv* – dorsal blood vessel; *gl* – pygidial glands; *in* – intestine; *lm* – ventral longitudinal muscle bands; *opm* – oblique pygidial muscles; *rpm* – radial pygidial muscles; *sc* – segmental coeloms; *vpm* – ventral trapezoidal transversal muscle; *vv* – ventral blood vessel. Scale bar 50 μm.

*P. dumerilii* possesses a complicated musculature in the pygidium. Staining with TRITC-phalloidin reveals that the dorsal and lateral surfaces of the pygidium contain a strong array of circular muscles (Figure 4B), playing the role of an anal sphincter. Similar circular muscles also exist in segments but they are much less developed. At the anterior border between pygidium and last body segment lays an additional thin circular muscle (Figure 4B, C). A number of thin radial muscular fibers are passing from this circular muscle to the gut lining (Figure 4C). At the ventral side in the anterior part of the pygidium is situated a short trapezoidal transverse muscle (Figure 4A). A pair of oblique muscles, connecting to the basis of the tentacular cirri is linked to its lateral sides.

### The pygidium contains a coelom-like cavity

**Figure 5:**
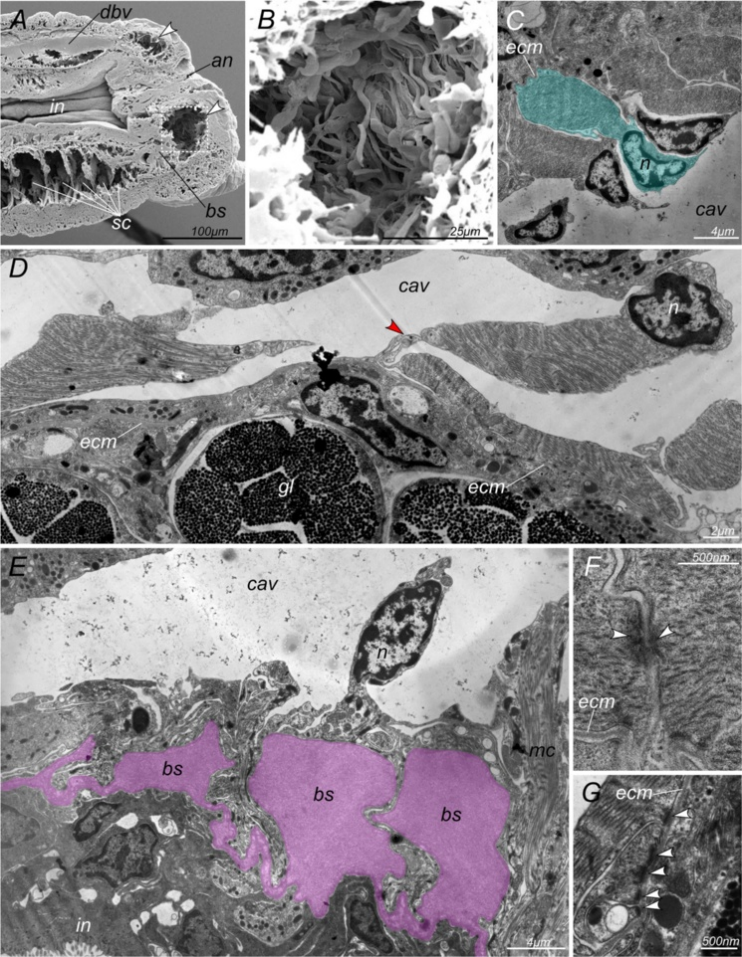
**Ultrastructure of** ***P. dumerilii*** **pygidial cavity inlay. A**: Scanning electron micrograph of sagittal dissection of posterior part of the worm showing pygidial cavity (arrowheads) and segmental coeloms (*sc*). **B**: Closer view of the ventral part of the pygidial cavity (rectangle selection in **A**) showing inlay cells. **C-G**: Transmission electron micrographs of different parts of the pygidial cavity inlay. **C-D**: Different kinds of myoepithelial cells, red arrowhead marks thin cytoplasmic lamella connecting two parts of the cell; **E**: visceral myoepithelium and blood vessels; **F**: Adherens junctions between myoepithelial cells (arrowheads). **G**: Hemidesmosomes, attaching myoepithelial cells to the basal membrane (arrowheads). *an* – anus; *bs* – perianal blood sinus; *cav* – pygidial cavity; *dbv* – dorsal blood vessel; *ecm* – extracellular membrane; *gl* – pygidial glands; *in* – intestine; *n* – cell nucleus; *sc* – segmental coeloms.

Confocal optical sections from TRITC-phalloidin stained animals show a vast “empty space” between pygidial body wall musculature and hindgut muscular lining (Figure 4D). Series of semithin (500 nm) resin sections reveal that this corresponds to a cavity inside the mature and newly formed pygidia (Figure 4E-G). This cavity appears also clearly in scanning electron microscopy (SEM, Figure 5A, B). Histological sections and scanning electron micrographs show the presence of a cellular lining around this cavity. 3D reconstructions, made by series of semithin sections clearly show that, unlike the segmental paired coelomic cavities, it is unpaired and has a torus-like shape (Figure 4H). We next used transmission electron microscopy (TEM) to identify the elements that would allow characterizing this pygidial cavity as a true coelom or not. We identified all of the elements of a typical mesodermal epithelium around this cavity, similar to the epithelia surrounding the segmental coelomic cavities. A monolayer epithelium lays on a basal lamina (Figure 5C-G), showing an apico-basal cell polarity. The cells possess hemidesmosomes in contact to the basal lamina and apical junctions (Figure 5F, G, arrowheads). The lining cells are in vast majority myoepithelial, containing well visible myofibrils (Figure 5C). They often take very convoluted shapes. Some cells seem at first glance to be floating freely inside the cavity but they remain connected to the epithelium by thin cytoplasmic lamellae (Figure 5D, red arrowhead). Also, in places, the basal lamina is only covered by a thin lamella of cytoplasm (Figure 5D). We do not find evidence of free-swimming cells in the pygidial cavity. We also did not find any signs of segmental organs.

### A circular blood sinus is located in the dissepiment between the pygidial cavity and the mesodermal addition zone

This contractile vessel is clearly visible on live individuals and on sections (*bs,* Figure 4E-H, Figure 5 E). It is immediately posterior to the SAZ and connected to the ventral and dorsal longitudinal vessels. The sinus is enclosed on one side by a basal lamina produced by myoepithelial cells and on the other side by the gut epithelium. It is therefore similar to the gut sinus present in segments but much more spacious. No blood vessel was found posterior to this sinus.

### Epitokous changes of the pygidium

**Figure 6:**
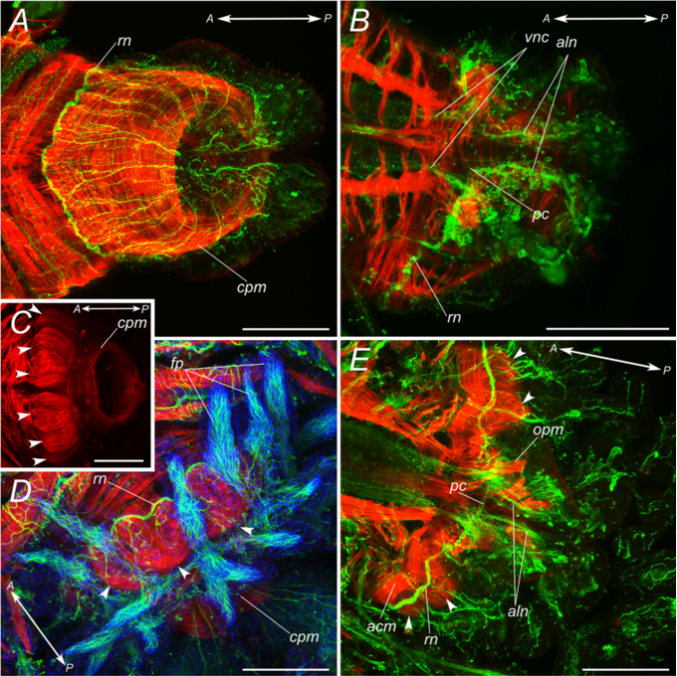
**Epitokous transformation of** ***P. dumerilii*** **pygidium.** Confocal micrographs, showing acetylated α-tubulin immunoreactivity (green), TRITC-phalloidin stained muscles (red), and cell nuclei (blue). **A-B**: Pygidia of epitokous females. **C-E**: Pygidia of epitokous males. **A, C, D** - view from the dorsal side; **B, E** - view from the ventral side. *vnc* - ventral nerve cord; *rn* - pygidial ring nerve; *pc* - pygidial commissure; *aln* - additional pygidial longitudinal nerves; *cpm* - circular pygidial muscles; *acm* - additional circular pygidial muscle; *opm* - oblique pygidial muscles; *fp* - finger-like protrusions of the male pygidium filled with spermatozoa. Arrowheads show newly formed small longitudinal muscles of the male pygidium. Scale bar 100 μm.

As it was noticed before, the pygidium is involved in the sets of transformations of epitokous metamorphosis (Figure 1D, E; Figure 6). While changes in females are limited, including an increase of the musculature (Figure 6A, B), in males transformations are far more significant. The surface of mature male pygidium bears finger-like protrusions filled with sperm that is clearly seen in confocal images (Figure 6D). Musculature also transforms. In the anterior border all around the pygidium a number of small longitudinal muscles appear (Figure 6C-E, arrowheads). This system probably works like a pump for controlled sperm release that is characteristic in *P. dumerilii*.

## Discussion

### The Structure of *P. dumerilii* pygidium is far more complicated than previously acknowledged

**Figure 7:**
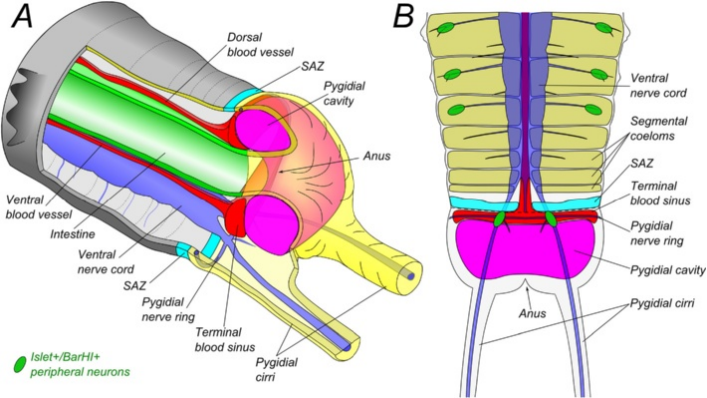
**Generalized schemes of the posterior region of** ***Platynereis dumerilii*** **. A**: view from the lateral side. Part of the body wall and segmental coeloms are not shown. **B**: view from the ventral side. SAZ shows the position of the ring of ectoteloblasts only. The underlying ring of mesoteloblasts is not shown for the sake of readability.

Despite a long and active history of polychaete anatomic studies, the pygidium has remained an understudied part of the annelid body. Here we provide the first detailed morphological description of the pygidium in *Platynereis dumerilii*. A general scheme of the pygidial region is presented in Figure 7. We have found complex intrinsic nervous system and musculature. Most importantly, the pygidium possesses a large torus-shaped cavity that in many ways is similar to the segmental coeloms but at the same time is characterized by a series of unique characters. Because of this pygidial coelom, combined with a number of neural and mesodermal characters we described above, can we call the annelid pygidium a “segment”? The problem of segmentation is complicated. Different authors propose their own definition of what constitutes a segment. Gerhard Scholtz [24] defined a segment as “an antero-posteriorly repeated body unit, which can be defined by a set of sub-structures or characters in a specific spatio-temporal correlation”. Clearly, these sub-structures vary between phyla. For annelid segments, Scholtz proposes: an outer annulus, one pair of mesodermal hollow spaces, one pair of ventral ganglia, one pair of nephridia, a set of muscles and one pair of appendages. The comparison of character states which are critical for the definition of a segment between pygidium and mid-body segments of *P. dumerilii* is summarized in Table 1. However, as we already mentioned in the introduction, the question of the nature of the pygidium cannot be treated only from the point of view of a “typological” definition. The resemblance of the pygidium to a trunk segment is a product of evolution and must be explained in this framework. According to recent phylogenies [16,19], the segment definition given by G. Scholtz probably corresponds to ancestral characters in annelids and constitutes a good basis to compare with pygidial characteristics. To this list, it seems to us crucial to add developmental characteristics. Developmental characteristics include structural characters that develop only during the larval stages, such as the ciliary belts. We also looked at some of the genes that are specifically involved in the formation of some structures, providing a “molecular signature” for neural progenitors. None of the characters presented above is by itself decisive in showing whether the pygidium may be related to segments. However, taken together, they can lead to the conclusion that the pygidium is nothing but a highly derived segment, adapted to its special functions.

**Table 1:** **The state of segment specific characters in mid-body segment and the pygidium in** ***P. dumerilii***

**Character**	**Body segment**	**Pygidium**
***Outer annulus***	Present	Present
**Appendages**	One pair of biramous parapodia with each two cirri and two chaetae bundles	One pair of cirri
***Ventral ganglia***	Paired ganglia interconnected by a commissure	Absent, but a pygidial commissure is present
***Peripheral ganglia***	One pair of parapodial ganglia	One pair of presumably cirral ganglia
***Muscular elements***	Longitudinal muscle bundles, transverse muscles, parapodial muscle complexes	Circular muscles, specific ventral muscle complex
***Blood vascular system***	Dorsal and ventral longitudinal vessels, lateral and parapodial vessels, gut plexus	Circular blood sinus
***Secondary cavity***	Paired coelomic sacs delineated by myoepithelial cells formed by schizocoely	Unpaired coelomic cavity delineated by myoepithelial cells, of unclear developmental origin
***Free swimming cells in the secondary cavity***	Numerous different coelomocytes	Absent
***Nephridia***	One pair of metanephridia	Absent

The detailed analysis of each described character is here required to assess whether the segmental version and the pygidial counterpart can be traced back to a single ancestral metameric sub-structure. We will start with the neural-associated characters followed by the mesodermal derivatives.

The telotroch is a transient structure associated with larval locomotion. It is described in a large number of annelid groups, but it is a characteristic worth discussing in relation with pygidial structure. In *Platynereis*, the telotroch is made of only two pairs of multiciliated cells (trochoblasts) on each side of the proctodeum. A telotroch is present in many other annelid groups and can be made of four to six cells organised in a ring around the pygidial area [22]. Telotroch cells are located posterior to the SAZ and are therefore pygidial by definition. Segmental ciliary belts known as paratrochs are a larval characteristic quite common among annelid families [35]. In *P. dumerilii*, only the three larval chaetigerous segments bear a paratroch but in species where a much larger number of larval segments are made, such as Spionidae, each one has a paratroch to propel the elongated larva into the water column. The last common ancestor of annelids probably had both a telotroch and paratrochs and it is conceivable that the telotroch is a serial homologue of the segmental paratrochs. It should be noted however that the telotroch is situated anteriorly in the pygidium whereas paratroch locations are usually interpreted as median or posterior in the segment.

The nervous system in the *Platynereis* original or regenerated pygidium possesses both a nerve ring and a thin commissure. In annelid larvae, ciliary belts are usually associated with a nerve ring. In *Platynereis* as well as in most annelid species, the segmental ring nerves grow ramifications that innervate parapodia later in development (Nezlin and Starunov, unpublished observations). The pygidial nerve ring that we describe in *Platynereis* has presumably grown from a telotrochal nerve ring. We could not see however a nerve ring associated with the telotroch in larvae (Figure 2). The absence of the ganglia of the main ventral nerve cord may be correlated to the absence of complete parapodial appendages in the pygidium. The pygidial commissure could be a remnant of segmental ganglia but, in the absence of a pygidial central nervous system, it cannot carry the axons of centrally located interneurons as annelid segmental commissures normally do. A similar commissure can be observed in illustrations by Müller [36], Müller and Westheide [37], Orrhage and Müller [38], and Müller and Henning [39], however in all these papers the authors just mark it as “terminal commissure” without any further discussion. Further immunohistochemical observations in broader range of annelid families are needed to define whether or not described nervous structures are common for annelids and represent a part of their groundplan. The ring nerve has presumably specific functions, such as innervating the hindgut sphincter and, possibly, the SAZ.

It is interesting to note that the first neurons in polychaetes appear in the region of the future pygidium [29,40-42]. It was suggested that these neurons have a morphogenetic function, their projections forming the scaffold of the future central nervous system [43,44]. They are transitory and cannot be distinguished in adult worms. Thus the future pygidial region of the larva may play an important role in early annelid neurodevelopment.

The pygidium of *P. dumerilii* bears two groups of presumed sensory neurons, located at the base of the tentacular cirri. The tentacular cirri of the pygidium look very similar to the tentacular cirri of the head (in fact modified parapodial cirri) in structure and size and to the parapodial cirri of the trunk in structure. The tentacular cirri of the head are supplied by small cirral ganglia [45] and each segmental parapodium also possesses its own parapodial ganglion [46] innervating the parapodial cirri. Likewise, two small ganglia are located near the base of the pygidial cirri and both the developmental molecular signature (*BarH1*/*islet* +) and the pattern of serotonin- and FMRFamide-like immunoreactivity found in the pygidium (FMRFamide-positive neurons and serotonin-positive neurite meshwork) are similar to those in parapodial ganglia making them possible serial homologues. It should be noted however that the pygidial ganglia are not well condensed whereas parapodial ganglia are cohesive groups of perikarya. The pygidial cirri and pygidial epidermis in *P. dumerilii* are covered with many receptor cells and free nerve endings (our SEM and WMISH observations), suggesting that the pygidium, far from being reduced to an anus-bearing piece, has well developed sensory functions. In other annelid groups, the sensory equipment of the pygidium is varied. Some groups have a simple pygidium with no cirri (Cirratuliformia for instance), many groups have a single or two pairs of pygidial cirri and a few have more than two pairs of cirri (some spionids for instance). In some sabellids, the pygidium even presents photoreceptor organs that are non-homologous to the eyes of the head [47]. Investigations on the nervous system of the species carrying two pairs of pygidial cirri would be useful to examine whether their pygidium is closer in organization to the typical annelid segment, carrying two pairs of parapodial cirri.

The muscular system found in the pygidium is elaborate and does not resemble closely the segmental musculature. Circular muscles act as hindgut sphincter. The anterior circular muscle and the radial fibers could regulate the activity of the posterior blood sinus. The ventral pygidial muscles may act to move the pygidial cirri. It is important to note that the head tentacular cirri possess a similar musculature. However, in the head, muscle fibers encircle the bases of cirri for greater motility. The presence of a circular musculature in the annelid ground pattern is a contentious issue [48,49]. In nereidids, circular or transverse muscles are poorly developed in the larva or in fully developed segments. Somatic circular muscles are however visible on our phalloidin stainings in developing segments, suggesting that a circular musculature develops in opposite directions in segments and in the pygidium; in segments, the forming circular muscles are subsequently lost or reduced to a few tranverse fibers whereas in the pygidium, they strengthen to give the anal sphincter and the anterior circular muscle. It would be important in this respect to investigate the pygidial musculature in different annelid species and compare it with the segmental musculature.

The pygidial circular blood sinus is a prominent structure. It is located in the immediate vicinity of the rings of ectodermal and mesodermal teloblasts of the SAZ. As the teloblasts are indeed small stem cells that produce all the tissues of the trunk of *Platynereis* [20], they presumably require a constant supply of nutrients, provided by the blood circulating in this lacuna. Another function may be hormonal signaling. In Nereidids, neurohormonal regulation of growth has been demonstrated [50-53]. Neurohormones are synthetized in an infracerebral gland and distributed through the blood vascular system. The large size of the pygidial blood lacuna may serve to maximize the contact surface both for better nutrients and neurohormone diffusion.

The pygidial cavity is structurally identical to the coelomic cavities of body segment. It is a true secondary body cavity lined by a myoepithelium and fits well the definition given by Bartolomaeus [54]. However it is unpaired, lacks segmental organs, circulating cells and it is not connected to the rest of the coelom. The blood vascular system terminates posteriorly at the anterior border of this pygidial coelomic cavity and does not extend further inside the pygidium. The pygidial coelom presumably works as a hydroskeleton, antagonistic to the anal sphincter. Another possible role is in gamete release in epitokous individuals, especially males.

Two different explanations for the origin of the pygidial cavity are possible and are both of highest evolutionary significance. The first scenario postulates that this cavity is a derived segmental coelom, transformed according to its special functions. In this case, the unpaired condition and lack of the segmental organs should be a derived condition. The second scenario would propose that the pygidial cavity has been formed *de novo* and does not represent a homologous structure to the segmental coeloms. The pygidial coelomic cavity has never been truly described as such in any annelid. In nereidids, it appears incidentally on a drawing of a posterior regenerate in *Nereis diversicolor*, but without any further explanation or description [55] and it was more recently briefly mentioned in another nereidid, *Alitta virens* [56]. It is missing in most classic descriptions of annelid larvae and juveniles. In the orbiniid *Scoloplos armiger*, for instance, Anderson [57] mentions a small, solid group of mesodermal cells around the hindgut (which he terms residual mesoderm) from which the newly formed somites bud off. The dominating view has been that the annelid pygidium contains no specific differentiated mesodermal tissues but some mesodermal stem cells responsible for posterior growth. Our observation contradicts this view at least in Nereididae. The stem cells responsible for the posterior addition of axial mesodermal tissues have been convincingly identified by molecular signatures [20]. This mesoteloblast population is located well in the anterior part of the pygidium, indicating that the rest of the pygidial mesoderm, posterior to teloblasts, is indeed made of intrinsically pygidial differentiated cells. We must insist that we did not observe any cavity in larvae and early juveniles and we do not know exactly when this coelomic cavity forms. This cavity thus arises late in development and may have been overlooked in other annelid species. We incidentally notice that structures like an undescribed cavity posterior to the segment addition zone are visible in the capitellid *Capitella teleta* [58] (in the Figure nine, panels C and F1; confirmed by a personal communication to ND) and in the clitellate *Enchytraeus* [59] (in the Figure three, panels K, L, M, “anal segment”). This probably shows that a pygidial coelom is a more widespread characteristic than one may think because these two species are only distantly related to *P. dumerilii* within annelids [17,19]. Further researches of pygidial organization and development will solve this problem.

In annelids, the pygidial ectoderm is derived from the same 2d blastomere as the segmental ectoderm is and its mesoderm from the same 4d blastomere [60]. We do not know exactly when the pygidial coelom forms but it is clearly after the larva has settled and started to grow new segments. Generally speaking, the pygidium forms late and progressively compared to the larval segments.

### Pygidium and metameric theories

There are a number of possible evolutionary interpretations to the elaborate set of structural similarities that we describe in this work between the pygidium and trunk segments. Interpretations can be divided in two categories: convergence or homology. The interpretations involving convergence postulate that the various structural elements that compose the pygidium have evolved under adaptative pressure to resemble those elements that are found on trunk segments. The functions performed by the pygidium (defecation, developmental elongation, gamete release) have however little in common with those performed by a trunk segment (locomotion, excretion, etc.…), making an interpretation based only on convergence unlikely.

The interpretations involving the homology of the structures described can be divided into two types, based on whether the annelid pygidium is implied to be either of non-segmental or segmental origin. The former case involves the assumption that the supposedly peculiar pygidium of *P. dumerilii* may have arisen in evolutionary history by the fusion of the ancestral non-segmental annelid pygidium and a reduced body segment. Segment fusion has been a common place event in annelid history. Such a situation takes place during the development of the Nereidid head region. The anterior-most larval segment does not form chaetae and parapodia and is fused early with the larval head [56,61]. Later in development, the first chaetigerous segment of the larva loses its parapodial lobes and chaetae and takes part in the formation of the peristomium. The four pairs of tentacular cirri of the head region derive directly from the cirri of these two anterior segments fused to the head. However, applying this sort of explanation to the posterior region leads to a major ontogenetic problem: a reduced body segment would be produced posteriorly to the SAZ of *P. dumerilii,* a phenomenon that has not been described in any annelid species. In particular, there is no evidence coming from *P. dumerilii* development in favor of a composite origin of the pygidium.

**Figure 8:**
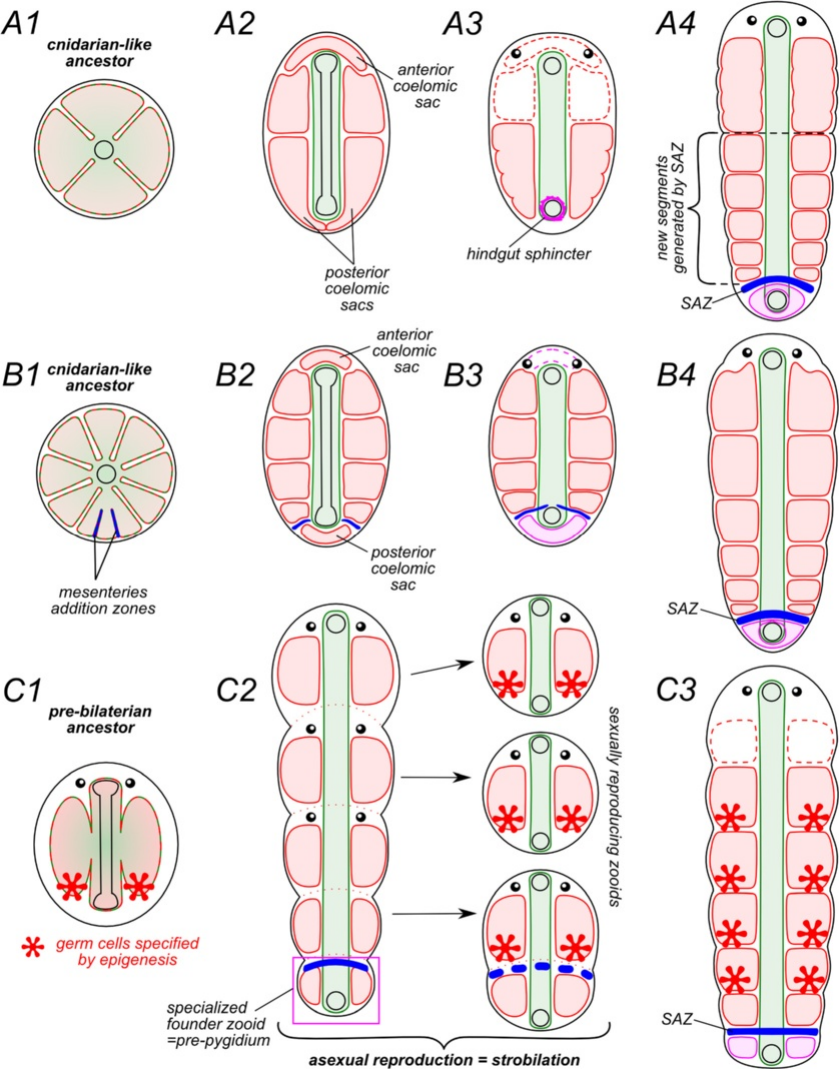
**Schematic diagrams describing three hypothetical evolutionary scenarios for the origin of the metamery and the pygidium.** The classic cyclomeric theory **(A1-4)** postulates that the hypothetical cnidarian-like ancestor came to creeping on its oral side. It primary mouth elongated along the newly formed antero-posterior axis. The gastric pouches were separated from the main digestive cavity, giving rise to coeloms. Then anterior coelomic sacs were reduced. The posterior coelom subdivided into two lateral sacs and the SAZ formed posterior to them. The pygidial coelom was formed from the hindgut sphincter by schizocoely and is not homologous to the segmental coeloms. We propose a variant of this cyclomeric hypothesis, more in accordance with the discovery of a true coelomic cavity in the pygidium **(B1-4)**. It suggests that the SAZ has evolved from a mesentery addition zone, located at the “posterior” part of the radial ancestor (as described in contemporary Ceriantharia [68]). The pygidial cavity was formed from unpaired gastric pocket located between two regions of mesentery addition. In the very different colonial theory **(C1-3)**, the pre-bilaterian metameric ancestor derives from a linear colony of sexually reproducing individuals or zooids, that is itself initially produced by asexual budding from a “founder” zooid. In this scenario, the pygidium is homologous to the body segments and represents the founder zooid remnant.

The last set of explanation involves a common origin of the similar neural and mesodermal structures of the pygidium and trunk segments. As such, it cannot be discussed without reference to the various theories on the origin of segmentation, one of the key issues in evolutionary biology. Since the nineteenth century, there are two main alternative theories: the cyclomeric (also called enterocoele or archicoelomate), and colonial theories. In the cyclomeric theory, occasionally described in textbooks, the serial organization is derived from the gut pouches of an initially radial organized, cnidarian-like ancestor. Those gut pouches evolving as a seriated coelom provide the basic scaffold on which a metameric organization can be built to give an annelid-like organism [62-65]. Every segment in this theory corresponds to a pair of opposite gastric pockets of the hypothetical ancestor (Figure 8A). In this “classic” cyclomeric theory, the pygidium derives from the tissues located in the vicinity of the “posterior” end of the elongating slit-like blastopore that will eventually give the anus. It does not provide however an explanation as to why the pygidium should resemble in any way the mid-body segments. The only possible way is to suppose that the pygidial cavity evolved from the hindgut sphincter by fluid accumulation (Figure 8A3-A4) [66]. As an alternative, we propose to complete this “classic” theory to account for the presence of a coelomic cavity, derived from a gut pouch(es) in the pygidium. There are two conceivable scenarios to explain the complex structure of the pygidium with an unpaired coelomic cavity with all other structures being paired: the first one would be that the future pygidium inherited two neighboring gastric pockets, as well as the associated neural and sensory organs, as did mid-body normal segments. The coelom then becomes secondarily unpaired later in evolution. The second possibility would be that the pygidium inherited an unpaired coelom and the unpaired associated organs (Figure 8B). This second possibility postulates an unusual pygidial condition with unpaired appendages. Although this condition is known in a small number of annelid groups [67], almost nothing is known about the innervation of these unpaired appendages. Further studies will shed light on this problem. In this cyclomeric-derived scenarios, the pygidium is not directly homologous to a body segment but has inherited a number of homologous structures in common with mid-body segments. Both the pygidium and prostomium are therefore not truly derived from the same original state as body segments, but share a common origin with them, being built around the gastric pockets of a cnidarian-like ancestor. This would explain the structural similarities they share with trunk segments. In addition and in this context, the SAZ may derive from a mesentery addition zone as described in some anthozoan cnidarians [68].

By contrast, the colonial theory (Figure 8C) accounts for the fact that the pygidium might be a derived structure homologous with body segments. The colonial theory essentially states that segmented organisms are derived from linear colonies of simply-built individuals (or zooids) produced by asexual reproduction [69-72]. The zooid chain is produced by sequential budding from a founder zooid, itself born from sexual reproduction. In consequence, the entire body of the resulting animal is formed from modified metameric units. The pygidium is the direct descendant of the founder zooid and as such is serially homologous with trunk segments. There are some difficulties with this theory. One objection is that, in the initial linear colony, the founder zooid buds off all the other zooids, while in annelids, the pygidium is always fully differentiated only after a variable species-specific number of leg-bearing larval segments (for instances, three segments in *P. dumerilii* but as many as thirteen in *Capitella teleta*, [73]) ([14] for a review). The theory also requires that the entire body of an annelid, including the head, be derived of segment-like units. The head section is very specialized, complex in its developmental origin and very variable between annelid groups. Investigations towards understanding what are the ancestral ‘building blocks” of the head in annelids are a key objective for the future.

Is there corresponding evidence coming from other groups in the bilaterian tree? In chordates, obviously, a proper pygidium does not exist as the anus is disconnected early in development from the tail bud growth zone of the embryo. In arthropods, the old Articulata concept homologizes the annelid pygidium with the arthropod telson. If segmentation is indeed ancestral in bilaterians or at least in protostomes [10,12], the pygidium/telson homology is a clear possibility. The telson has evolved differently in different arthropod groups and is sometimes so reduced in the adult that what remains of it is equivocal. This is essentially what has happened in groups (such as insects) where the ancestral post-embryonic segment addition has been replaced by embryonic formation of the whole complement of segments. In crustaceans still showing post-embryonic posterior addition of segments, such as *Artemia* [74], a distinct telson is visible posterior to the segment addition zone and actually grows *distal-less* expressing appendages in late development. This telson and its contiguous growth zone, like its annelid counterpart, express the genes *caudal* and *even-skipped*. However, no coelomic compartment is ever associated with the telson in any arthropod. In onychophorans, the anus is found on an “ultimate segment” that bears no legs but has coelomic cavities and actually develops glands derived from nephridial anlagen [75]. This piece of the body could be interpreted as a pygidium if our hypothesis is true. Alternatively, the pygidium could also be reduced to the hindgut.

## Conclusions

The pygidial region in *Platynereis dumerilii* is far more complicated than usually mentioned in annelids. The pygidium possesses a complex nervous system with its own peripheral ganglia, a complex musculature and a cavity that can be identified as a true coelomic cavity. Such a complex structure indicates that the pygidium plays an important role in the life of the animal. It acts as a SAZ-associated structure, presumably regulating its activity, as a sensory structure as well as a hindgut sphincter. Furthermore, a specific role of the pygidium in male mating behavior and reproduction was shown.

Our results contradict one aspect of the classical consensus on the unsegmented nature of body ends in annelids and provide new material for evolutionary considerations about the origin of bilaterian body plan and segmentation. More comparative studies are needed to analyse the distribution of this unusual organization among other annelids, its origin and functions. A key objective for the future would be to discover similar pygidial coelomic cavities in other annelid families, which would suggest that this pygidial coelom is an annelid ancestral character and not a nereidid autapomorphy. The development of the pygidium should also be investigated in the future. If a common origin with trunk segments is true, one can expect that at least a part of the genes and signaling pathways that are used for the early embryonic and larval patterning of segments and pygidium will be similar. Another key developmental issue is the compared cell lineages of the pygidium and segments in the annelid embryo. Do these lineages share similar elements that would support homology? Last, we have to establish when and how the pygidial cavity is actually formed in *P. dumerilii.* Does the pygidial coelom arise from paired mesodermal pockets that later fuse to form an unpaired cavity? We expect that these future studies will contribute to our understanding of the evolution of annelid segmentation and provide new strong evidences to any of the segmentation origin theories described above.

## Methods

### Animals

*Platynereis* specimens were obtained from laboratory cultures at Institute Jacques Monod, CNRS, France and Saint-Petersburg University, Russia. Animals were dissected by razor blade and fixed in 4% paraformaldehyde in phosphate buffered saline (PBS) overnight at 4°C then washed three times in PBS and stored in PBS, containing 0,1% NaN_3_ at 4°C. Animal experimentation was carried out according to international and national ethics guidelines, under the scrutiny of the local ethics committee (*CEEA40-Comité d'éthique Buffon).* Agreement for animal care (ref. #A75-13-17-2) is delivered by the Direction Départementale de la Protection des Populations de Paris.

### Semithin sections and electron microscopy

For transmission electron microscopic studies animals were fixed 2 hours in 2,5% glutaraldehyde in 0.1 M cacodylate buffer, washed in cacodylate buffer (3 × 15 minutes) and postfixed in 0.1% OsO_4_ (2 h) in the same buffer. To normalize the osmotic pressure, corresponding amount of sucrose were added to all EM fixatives and washing buffers. Specimens were dehydrated in a graded series of ethanol and embedded in EMbed-812 (Electron Microscopy Sciences, 14120) or Araldite-EMbed 812 (Electron Microscopy Sciences, 13940).

Series of semithin (500 nm) sections were made using Leica-Reichert Ultracut S ultramicrotome and Diatome Histo Jumbo diamond knife [76]. Sections were stained with toluidine blue or methylene blue-azurB-fuchsin [77], scanned using Olympus VS120 imaging system and aligned with Imod (imodtkalign plugin, [78]). 3D reconstructions were made using TrackEM2 plugin for FIJI [79]. Virtual sections were made with Imaris (Bitplane, Zürich, Switzerland).

Ultrathin sections were made using Leica UC7 ultramicrotome with diamond knife, stained with uranyl acetate and lead citrate in Leica EM AC20 staining device and examined at Jeol JEM-1400 transmission electron microscope.

For scanning Electron microscopy specimens after glutaraldehyde fixation were dehydrated in alcohol, immersed in acetone, critical point dried and carbon coated. Specimens were examined using Tescan MIRA3 LMU scanning microscope.

### Immunolabelling

Formaldehyde fixed specimens were washed in PBS, containing 0.1% Triton X-100 (PBT), followed by overnight incubation in blocking buffer (1% BSA in PBT). Then specimens were incubated in primary antibodies against acetylated α-tubulin (Sigma, T-6793, produced in mouse) and serotonin (Immunostar, 20080, produced in rabbit) or FMRFamide (Immunostar, 20091, produced in rabbit) diluted 1:1000 in PBT for 27-72 h. Subsequently specimens were washed 3x15 minutes in PBT and incubated with secondary antibodies overnight. We used the following secondary antibodies diluted 1:1000 in PBT: Alexa Fluor 488 donkey anti-rabbit, A-21206, Invitrogen; Alexa Fluor 647 donkey anti-mouse, A-31573, Invitrogen; CF633 goat anti-mouse, SAB4600333, Sigma; CF555 goat anti-rabbit, SAB4600300, Sigma. After secondary antibody incubation, specimens were stained with TRITC-conjugated phalloidin (Molecular Probes, R415) diluted 1:100 in PBT for 2 h, followed by DAPI counterstaining. All the incubations were performed at 4°C. Finally specimens were gradually immersed in 80% glycerol and mounted between two coverslips. Specimens were examined using laser confocal microscope Leica TCS SP5 or TCS SPE. The Z-stacks were projected using FIJI software. Final adjustments (brightness and contrast, levels or curves) were made in Adobe Photoshop.

### Gene cloning and visualization of expression patterns by Whole Mount In situ Hybridization (WMISH)

Genes were either already available from Genbank or found by homology in transcriptomes from the *Platynereis* resources database ([80], D. Arendt, personal communication). Accessions numbers for *Pdu-elav*, *Pdu-Hb9*, *Pdu-islet*, *Pdu-NK1*, *Pdu-Coe* and *Pdu-BarH1*, are [Genbank: EF384209, EF384221, EF384222, AM114772, GU169416, KP281292], respectively. DNA fragments approximately 1 kb long were cloned into PCRII plasmids using the TOPO cloning kit (Invitrogen). RNA probes were made and WMISH performed according using methods previously published protocols [21].
